# Multivariate Classification of *Prunus Dulcis* Varieties using Leaves of Nursery Plants and Near Infrared Spectroscopy

**DOI:** 10.1038/s41598-019-56274-5

**Published:** 2019-12-24

**Authors:** Sergio Borraz-Martínez, Joan Simó, Anna Gras, Mariàngela Mestre, Ricard Boqué

**Affiliations:** 1 Dept. of Agri-Food Engineering and Biotechnology, Universitat Politècnica de Catalunya, Campus Baix Llobregat, Esteve Terrades 8, 08860 Castelldefels, Spain; 2 Center of Initial Materials, Agromillora Iberia S.L.U, Ctra. BV-2247 km. 3, 08770 Sant Sadurní d’Anoia, Spain; 3 Universitat Rovira i Virgili, Dept. of Analytical Chemistry and Organic Chemistry, Campus Sescelades, 43007 Tarragona, Spain; 4 Fundació Miquel Agustí, Campus Baix Llobregat, Esteve Terrades 8, 08860 Castelldefels, Spain

**Keywords:** Statistics, Plant sciences, Infrared spectroscopy, Scientific data

## Abstract

The emergence of new almond tree (*Prunus dulcis*) varieties with agricultural interest is forcing the nursery plant industry to establish quality systems to keep varietal purity in the production stage. The aim of this study is to assess the capability of near-infrared spectroscopy (NIRS) to classify different *Prunus dulcis* varieties as an alternative to more expensive methods. Fresh and dried-powdered leaves of six different varieties of almond trees of commercial interest (*Avijor*, *Guara*, *Isabelona*, *Marta*, *Pentacebas* and *Soleta*) were used. The most important variables to discriminate between these varieties were studied through of three scientifically accepted indicators (Variable importance in projection¸ selectivity ratio and vector of the regression coefficients). The results showed that the 7000 to 4000 cm^−1^ range contains the most useful variables, which allowed to decrease the complexity of the data set. Concerning to the classification models, a high percentage of correct classifications (90–100%) was obtained, where dried-powdered leaves showed better results than fresh leaves. However, the classification rate of both kinds of leaves evidences the capacity of the near-infrared spectroscopy to discriminate *Prunus dulcis* varieties. We demonstrate with these results the capability of the NIRS technology as a quality control tool in nursery plant industry.

## Introduction

The almond market is in expansion. The world production in the 2017/2018 season reached the record of 1.3 million tons, 6% above the registered in the previous season, according to the latest estimates of the United States Department of Agriculture (USDA)^1^. The Food and Agriculture Organization Corporate Statistical Database (FAOSTAT)^2^ shows that, in Spain, in the period 2015–2017, the almond tree harvested area increased by 15%, which was reflected in an increase in production of 21%. These data demonstrate the importance of almond tree in world agriculture. For this reason, new varieties of almond trees have been increasing^3,4^. Varietal control in the production stage is a great challenge. The appearance of varietal mixtures within a batch, which should be homogeneous, is an important trouble, not only because the customer receives unwanted vegetal material, but also because nursery plant companies may face expensive fines and the deterioration of their corporative image.

The current method used for the identification of plants consists of performing molecular studies of the vegetal material in order to obtain molecular profiles and identifying the variety by using microsatellites and single nucleotide polymorphisms (SNPs)^5^. These biomolecular techniques are reliable. However, they are expensive and time consuming, and therefore cannot be applied routinely and with a high sample throughput. For this reason, the most common varietal control system consists of ensuring a correct and detailed traceability from the origin of the plant to its delivery to the customer, which is not always reliable. In this context, near-infrared spectroscopy (NIRS), combined with chemometrics techniques, is potentially a rapid, accurate, and non-destructive alternative.

In the last years, NIRS has gained importance in the agriculture sector as an interesting tool for monitoring and quality assessment of agricultural products^6–8^. Various studies have been published concerning the geographic origin^9–11^ and species discrimination in grapevine^12^, tea^13^, tomato^14^ and coffee^15^.

Moreover, several studies have shown the existence of an empirical relationship between leaf spectral properties and leaf physiological conditions^16,17^.

In the present study, six varieties of *Prunus dulcis* of agricultural interest were chosen: *Avijor*, *Guara*, *Isabelona*, *Marta*, *Pentacebas* and *Soleta*. All of them are genetically close because they come from crosses of traditional varieties, and some of them even share parents. In fact, this is the case of *Avijor* and *Marta*, both coming from the cross between “*Ferragnès x Tuono”*, and *Isabelona* and *Soleta* come from the cross between *“Blanquerna x Bella d’Aurons”*. Due to their genetic proximity, the morphology of these six varieties is difficult to distinguish.

Despite the present study focuses on specific varieties of *Prunus dulcis*, as the leaves’ composition is similar in all the angiosperm species^18^, the knowledge achieved in this work could be applied to discriminate other plant species.

The present study is the continuation of a previous research, where a sampling methodology was developed and optimized to analyze leaves from *Prunus dulcis* varieties using near infrared spectroscopy^19^. In the cited study, several sources of variability affecting the measurements were investigated, such as the regions of the leaves analyzed and the age of the leaves, and how these factors affect to the spectral signature of the varieties. Moreover, the most suitable preprocessing of the leaves and the best spectral pre-treatment were determined. In the present study, the information gathered from the previous research was used to assess the potential of NIRS to classify several varieties of almond trees (*Prunus dulcis*) that are genetically close and morphologically not distinguishable. The specific objectives are to: 1) develop a classification model capable of discriminating between six varieties of *Prunus dulcis*; 2) investigate the most important variables for the discrimination of the varieties; and 3) compare the classification results obtained with dried-ground leaves and fresh leaves.

## Material and Methods

### Description of the sampling field

The studied almond trees belong to the mother-plant field of the Centre of Initial Materials of Agromillora Iberia S.L.U., which is located in Sant Sadurní d’Anoia (Catalonia, Spain). The sanitary quality of the sampled trees was verified by means of molecular analysis, which Agromillora S.L.U. perform periodically as a quality control. Moreover, the trees were certified by the company. The same mother-plant field was used in a previously study^19^.

### Sampling protocol

Six *Prunus dulcis* varieties of agricultural interest were used. One hundred leaves were collected from ten trees for each variety. In total, six hundred leaves were collected from which three hundred fresh leaves were analyzed without any prior treatment, and the rest was dried and ground. The number of samples per variety was identical. Table 1 details the samples used in this study. The leaf samples collected were introduced in plastic bags with an identification code and stored at 4 °C until they were analyzed.

**Table 1:** Description of the different varieties of Prunus dulcis studied.

Varieties	Parents	Breeder	No. of fresh leaves	No. of dried-ground leaves	Harvesting time
*Avijor*	Ferragnès x Tuono	INRA	50	50	October 2018
*Guara*	Unknown	CITA	50	50	October 2018
*Isabelona*	Blanquerna x Bella d’Aurons	CITA	50	50	October 2018
*Marta*	Ferragnès x Tuono	CEBAS-CSIC	50	50	October 2018
*Pentacebas*	S5133 x Lauran	CEBAS-CSIC	50	50	October 2018
*Soleta*	Blanquerna x Bella d’Aurons	CITA	50	50	October 2018
Total			300	300	

(INRA = Institut National de la Recherche Agronomique (France); CITA = Centro de Investigación y Tecnología Agroalimentaria de Aragón (Spain); CEBAS-CSIC = Centro de Edafología y Biología Aplicada del Segura (Spain)).

### Sample pre-processing

To obtain dried-powdered leaves, fresh leaves were heated in an oven at 65 °C for 48 h. Once dried, a grinder was used to obtain a homogeneous powder. Further, the powder was stored in a desiccator with silica gel to avoid moisture^19^.

### Acquisition of NIR spectra

The NIR spectra acquisition method shown below was developed in a previous study^19^. An Antaris II FT-NIR analyzer (Thermo Scientific, USA), equipped with an integrating sphere module was used to scan the samples, which were measured in the spectral range of 12000–3800 cm^−1^ (833–2630 nm). The instrument configuration used consisted in the average of 32 scans by spectrum with a resolution of 4 cm^−1^. Each sample was analyzed in triplicate and the average of the replicates was used in the subsequent discrimination models. Every 20 minutes a background spectrum was collected. The powdered leaf samples were measured in a standard sample cup available with the instrument and fresh leaves were placed directly over the sphere. In both cases, samples were covered to prevent interference from environmental light. The reflectance spectra were mathematically transformed to absorbance by means of the log(1/R), where R is the reflectance. The room temperature was maintained at ~25 °C, and the humidity remained constant throughout the spectral acquisition process.

### Data preparation

When applying supervised multivariate classification methods, it is important to validate mathematical models by using an independent test data set. We applied two difference strategies to split the original dataset into calibration and test set: random split and the Kennard–Stone algorithm^20^. Similar results were obtained, so finally we decided to use the Kennard-Stone algorithm, which was applied to each class separately to split the data set into calibration set (70% of the leaves) and a test set (30% of the leaves).

### Spectral data pre-treatment

To enhance the spectral features and reduce systematic noise, such as baseline variation, light scattering, and path length differences, a mathematical pre-processing of the original spectra was necessary. The spectral pretreatment was optimized in a previous study^19^ and consisted of a combination of standard normal variate (SNV) with *Savitzky–Golay* (SG) first (1^st^) derivative filter and mean centering. SNV is a normalization procedure for spectral light scattering correction. It is used to correct additive and multiplicative effects in spectra caused by particle size variation. SNV calculates the standard deviation of all the variables in a given sample spectrum. The entire data set is then normalized by this value, which yields a unit standard deviation (*s* = 1) for the sample spectrum^19,21^. SG first derivative was applied to remove the baseline drift and enhance small spectral differences. The SG derivative method includes a smoothing step, the SG algorithm, which corrects the additional noise caused by the application of the derivative. The SG algorithm requires the selection of the order of the polynomial, order of the derivative, and filter width, which corresponds to the size of the window^19,22^. Herein, a 15-point window and second order polynomial were selected. Finally, mean centering was applied, which consists of calculating the mean value of each column and subtracting it from each individual value in the column. After mean centering, the mean value of each column equals zero, and each row of mean-centered data reflects only how it differs from the average sample in the original data matrix^19,23^.

The PLS_Toolbox (Eigenvector Research Incorporated, Manson, USA) with MATLAB R2017b (MathWorks, Natick, USA) were used to perform the spectral pre-treatments.

### Partial Least-Squares discriminant analysis (PLS-DA)

PLS-DA^24^ is based on the PLS regression algorithm and seek to find the linear combinations of the original variables (latent variables (LVs)) that have maximum covariance with the Y-variables (classes)^24^. Unlike PLS regression, in PLS-DA the Y-block is coded with dummy variables. Each sample is codified with one (1) if the sample belongs to a given class, and zero (0) if the sample does not belong to a given class. The result of a PLS-DA model is a series of prediction values for the different classes, that is, values around one for the class of interest and values around zero for the rest of classes. Finally, a threshold is calculated and optimized that optimally discriminates the different classes. Whit this PLS-DA model, validated with the test set of samples, it is then possible to predict the class of an unknown sample. In our case, six classes of leaves, one for each variety, were used, and therefore, the Y-block contained six columns.

A Venetian blinds cross-validation, with a data split of 10 and one sample per blind (thickness), was used to find the optimal number of factors (LVs) for the PLS-DA model^19^. The number of factors that showed the lowest classification error was selected as optimal. After the internal validation (optimization), the prediction ability of the model was assessed using samples that were not included in the calibration (30% of the total data set). The results are shown in Tables 2 and 3 (test-set validation).

To study the model results three different statistical parameters were calculated: sensitivity, specificity and accuracy. The sensitivity measures the proportion of actual positives that are correctly identified as such, the specificity measures the proportion of actual negatives that are correctly identified as such, and the accuracy measures the proportion of correctly classified samples, that is, the sum of true positives and true negatives divided by the total number of samples.

### Study of the important variables

The prediction accuracy of the full-spectrum PLS-DA model is negatively affected by the water absorption and other unrelated or collinear spectral variables. By selecting the optimal wavelengths, it is possible to reduce the complexity of the multivariate calibration model, by reducing the computational requirements and avoiding the inclusion of noisy variables. Spectral variable selection is then essential to generate models with better prediction accuracies^25^. However, it is crucial to use different variable selection methods in order to ensure that no important variables are eliminated.

The variable importance in projection (VIP) score, selectivity ratio and vector of regression coefficients were used to study the most important variables required to discriminate between the classes.

The VIP score is a parameter used for calculating the cumulative values of the influence of individual X-variables in the model^26^. In other words, VIP scores estimate the importance of each variable in the projection used in a PLS model^27^. Scores close to or greater than 1 are considered relevant.

The selectivity ratio provides a simple numerical assessment of the importance of each variable in a regression model^28^. By using the y-vector as a target, it is possible to transform the PLS components to obtain a single predictive target-projected component analogously to the predictive component in the orthogonal partial least squares discriminant analysis (OPLS-DA). By calculating the ratio between the explained and residual variance of the spectral variables on the target-projected component, a selectivity ratio plot is obtained and can be used for variable selection^29^. The larger the selectivity ratio, the more useful a variable for prediction. Variables with lower selectivity ratios may be excluded without degrading the performance of the model^28^.

The vector of the regression coefficients is an informative tool to select variables in multivariate calibration. The regression vector obtained from a multivariate regression can be defined as the expected change in the response per unit change in the variable if all the variables and responses are linearly related^30,31^. Variables with low regression coefficients do not contribute significantly to the prediction and can be removed.

## Results and Discussion

### Spectra investigation

**Figure 1:**
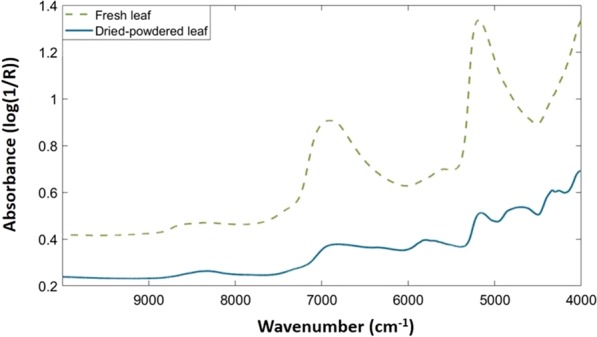
NIR mean raw spectra of fresh (green dashed line) and dried-powdered (blue solid line) leaves.

Figure 1 shows the mean raw spectra of the fresh and dried-powdered leaves. It can be observed that the shape of the spectra from both types of samples differs. This is due to the water content, which generates broad bands in the NIR spectra. However, both types of spectra present a low information zone that corresponds to the region between 7500 and 10000 cm^−1^. In contrast, the remaining region, from 4000 to 7500 cm^−1^, seems to be more informative, and the importance of this region will be evaluated below. It is noted that both regions were also observed in others studies performed on tea and bamboo leaves^13,32^.

By using NIR tables, it was possible to identify the main chemical groups behind the different bands. These bands in the NIR region correspond to the overtones and combination bands.

Regarding fresh leaves, two main broad bands were observed at 5000 and 7000 cm^−1^, corresponding to O-H vibrations and more specifically the combination O-H stretching and first overtone O-H stretching, respectively. Water is the main responsible of these bands as observed in a study developed on grapevine leaves^33^, although the O-H bond is also found in carbohydrates. Another important peak is observed between 4000 and 4500 cm^−1^. This absorption is attributed to the combination C-H stretching. The C-H bond is present in monosaccharides and, together with oxygen, conforms the aldehyde group, which is also present in carbohydrates.

Concerning the dried-powdered leaves, the range between 4000 to 7500 cm^−1^ includes the main bands, which correspond to the combination of C-H (7692–7042 cm^−1^), N-H (5000–4545 cm^−1^), and O-H stretching (5000–4545 cm^−1^) and the first overtone of C-H (6061–5556 cm^−1^), N-H and O-H (7143–6250 cm^−1^) stretching. All the bonds mentioned before are commonly found in carbohydrates and proteins. In addition, a small band can be observed between 8000 and 9000 cm^−1^ approximately. This band correspond to the second overtone of the C-H stretching (9091–8163 cm^−1^).

### Classification model using the whole spectra

Table 2 presents the results of the classification models for fresh and dried-powdered leaves. Concerning the dried-powdered leaf results, the classification accuracy in the cross-validation and test set validation was greater than 95% for all the varieties. In most cases, the test set validation showed a higher classification accuracy than that of cross-validation, except for *Avijor class*, due to the erroneous assignation of two *Guara* samples. This is not a common situation, as in general, better accuracy is reached for cross-validation than for test-set validation. However, sometimes this can happen when the test set does not contain a high number of samples. *Avijor* was presented the highest results for the cross-validation test, with a classification accuracy of 99.0%. In case of the test set validation, the best classification rate was obtained for the *Marta class*, with a classification accuracy of 100%. The lowest classification rate was obtained for *Guara*, which was the only variety showing an accuracy below 97%. Three *Guara* samples were misclassified in the test set validation, two samples were classified in the *Avijor class*, and one sample was assigned to the *Isabelona class*. Errors in the assignation of *Guara* samples were also reported in the cross-validation. Consequently, the *Guara class* showed a low sensitivity in both data sets. *Isabelona* and *Marta* showed the same sensitivity in both, cross-validation and test set validation. In addition, a correct assignation of all the samples of the validation data set was achieved for both varieties. *Marta* showed a higher specificity than that of *Pentacebas* because one *Guara* sample and one *Pentacebas* sample were misclassified in the *Isabelona class*. Thus, *Marta* presented better classification accuracy than that of *Isabelona* (100% and 97.8% accuracy, respectively). *Soleta* and *Pentacebas* showed the same sensitivity in the test set validation. The specificity was higher for *Soleta* than that of *Pentacebas* because one *Soleta* sample was erroneously assigned to *Pentacebas*, which was also reflected in a higher classification accuracy for *Soleta*.

**Table 2:** PLS-DA results of the classification of six varieties of *Prunus dulcis* using the entire spectra.

Real class	Data set	Sensitivity	Specificity	Accuracy
**Dried-powdered leaves**				
*Avijor*	Cross-validation	0.971	0.994	99.0%
	**Test set Validation**	**1.000**	**0.973**	**97.8%**
*Guara*	Cross-validation	0.829	0.983	95.7%
	**Test set Validation**	**0.800**	**1.000**	**96.7%**
*Isabelona*	Cross-validation	0.971	0.960	96.2%
	**Test set Validation**	**1.000**	**0.973**	**97.8%**
*Marta*	Cross-validation	0.971	0.983	98.1%
	**Test set Validation**	**1.000**	**1.000**	**100%**
*Pentacebas*	Cross-validation	0.886	0.989	97.1%
	**Test set Validation**	**0.933**	**0.987**	**97.8%**
*Soleta*	Cross-validation	0.886	0.994	97.6%
	**Test set Validation**	**0.933**	**1.000**	**98.9%**
**Fresh leaves**				
*Avijor*	Cross-validation	0.857	0.977	95.7%
	**Test set Validation**	**1.000**	**1.000**	**100%**
*Guara*	Cross-validation	0.857	0.943	92.9%
	**Test set Validation**	**0.600**	**0.987**	**92.2%**
*Isabelona*	Cross-validation	0.657	0.943	89.5%
	**Test set Validation**	**0.867**	**0.933**	**92.2%**
*Marta*	Cross-validation	0.943	0.994	98.6%
	**Test set Validation**	**1.000**	**1.000**	**100%**
*Pentacebas*	Cross-validation	0.914	0.983	97.1%
	**Test set Validation**	**1.000**	**1.000**	**100%**
*Soleta*	Cross-validation	0.743	0.954	91.9%
	**Test set Validation**	**0.933**	**0.960**	**95.6%**

Concerning the fresh leaf results, except for *Isabelona*, the rest of the varieties showed a classification accuracy higher than 90% in both cross-validation and test set validation. Specifically, an accuracy between 89.5–98.6% and between 92.2–100% was obtained fir the cross-validation and test set validation, respectively. The best classification results were obtained for *Marta* variety, with 98.6% and 100% of accuracy for the cross-validation and test set validation, respectively. *Pentacebas* and *Avijor* reached an accuracy of 100% in the test set validation. *Marta*, *Pentacebas* and *Avijor* presented a relatively low sensitivity in the cross-validation, but the samples were correctly predicted in test set validation. The lowest classification accuracy was obtained for *Isabelona* and *Guara*. *Isabelona* had a 89.5% accuracy in the cross-validation and 92.2% in the test set validation. *Guara* was the only variety showing a lower accuracy in the test set validation than that in the cross-validation. Four samples of *Guara* were assigned to *Isabelona*, and one sample of *Isabelona* was predicted as *Guara*. Similarly, in the dried-powdered leaf model, the *Guara* class was not correctly modelled. *Soleta* showed a high sensitivity in test set validation as only one sample of *Guara* was misclassified. However, due to the erroneous assignment of two *Guara* samples and one *Isabelona* sample, the classification accuracy of *Soleta* class was reduced. The highest specificity was obtained for *Marta*, *Pentacebas*, and *Avijor*.

For both models the prediction accuracy was high despite the genetic proximity of the varieties. However, the dried-powdered leaf model presented a higher accuracy in both the cross-validation and test set validation than that of the fresh leaf model. The homogeneity, and especially, the absence of water in the dried-powdered leaves seem to explain these results. The vibration of the water molecules generates broad bands in the near-infrared range that can hide other features, as it can be seen in the Fig. 1 and in other studies^34^. However, in of fresh leaf model, three varieties reached a 100% classification accuracy in the test set validation, in contrast, this was only achieved for one variety in the dried-powdered model. *Guara* was the worst classified variety in both models. In contrast, *Marta* showed a high classification accuracy in both models.

The full-spectrum PLS-DA model results were used as a reference to check that the variable selection process was correctly performed.

### Study of the most important variables

**Figure 2:**
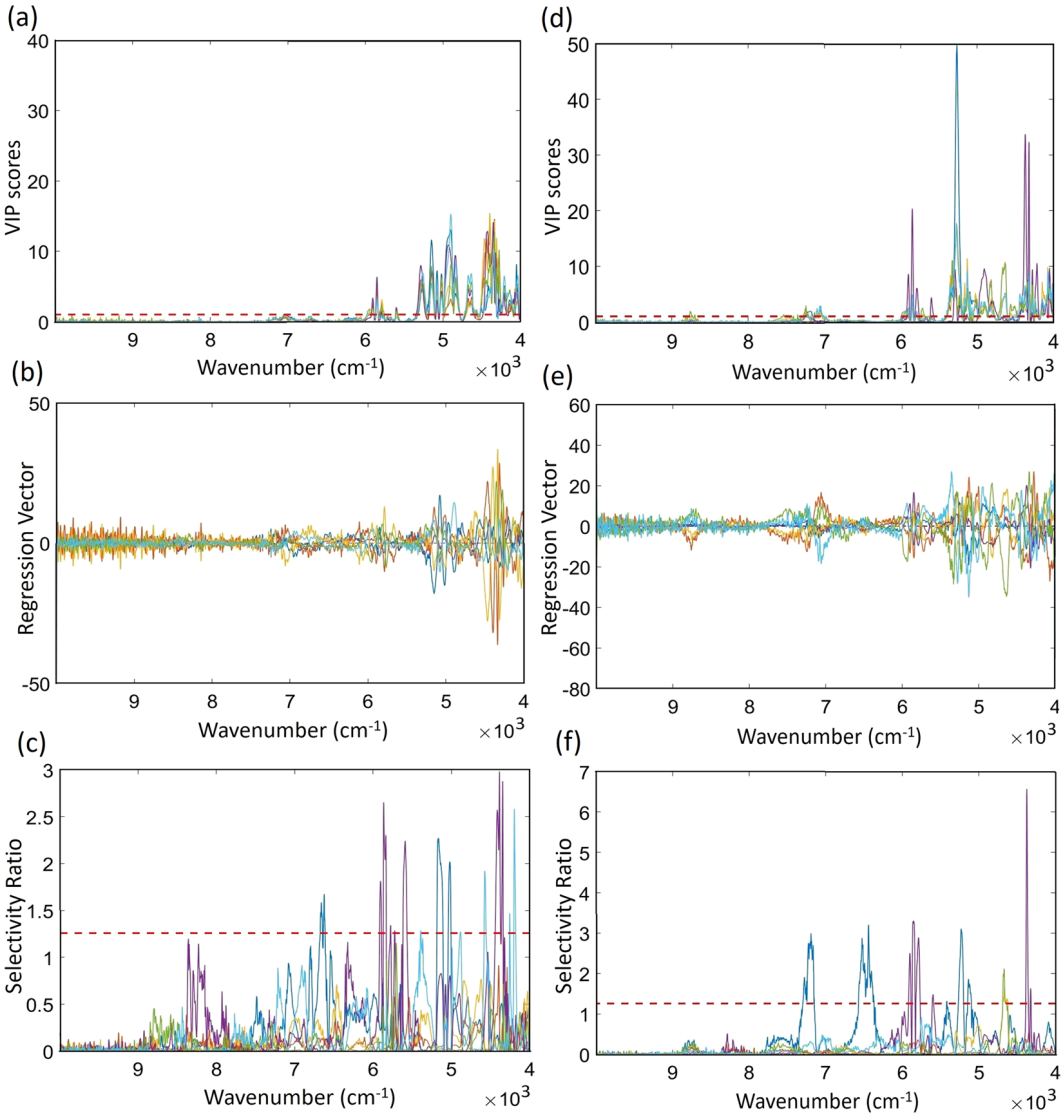
Variables selected for the dried-powdered and fresh leaves models. (**a**) VIP score of the dried-powdered leaves, (**b**) regression vector of the dried-powdered leaves, (**c**) selectivity ratio of the dried-powdered leaves (**d**) VIP score of the fresh leaves, (**e**) Regression vector of the fresh leaves, and (**f**) Selectivity ratio of the fresh leaves.

The VIP score, selectivity ratio, and vector of the regression coefficients were used to study the most important variables to discriminate between the classes. The results of these indicators are shown in Fig. 2.

Regarding the dried-powdered leaf model, the VIP score (Fig. 2a) showed important variables located in the range from 4000 to 6000 cm^−1^. Similar results were obtained with the regression vector (Fig. 2b). However, around 6600 cm^−1^, other important variables appeared in the selectivity ratio plot (Fig. 2c). In the range from 10000 to 7000 cm^−1^, no important variable was detected. Therefore, it was decided to cut the spectra from 6700 cm^−1^ in order to include only relevant variables.

Concerning the fresh leaf model, the VIP score and regression vector (Fig. 2d,e) showed important variables in the range between 6000 and 4000 cm^−1^. A small gap was detected between 7000 and 6000 cm^−1^, and the variables inside this range did not help the discriminate study. However, around 7000 cm^−1^, important variables can be found. The same results were obtained for the selectivity ratio indicator (Fig. 2f), except for the appearance of a band between 7000 and 6000 cm^−1^. The rest of variables were not useful to discriminate the almond tree varieties. Considering this, the fresh leaf spectra were cut from 7500 cm^−1^.

In both cases, fresh and dried-powdered leaves, the variables selected contain the bands related to C-H, O-H and N-H bonds, which can mainly be associated to proteins and carbohydrates, and water too in the fresh leaf. The amount of these bonds and their configuration inside the molecules affect the shape of the spectrum and, therefore, have an influence in the spectral fingerprint of the varieties. However, the region removed corresponded to wavenumbers with no important bands in the raw spectra (Fig. 1).

### Model using the most important wavenumbers

A new PLS-DA model was created using only the most important variables selected in the previous section. Table 3 shows the discrimination model results for dried-powdered and fresh leaves.

**Table 3:** PLS-DA results of the classification of six varieties of *Prunus dulcis* after variable selection.

Real class	Data set	Sensitivity	Specificity	Accuracy
**Dried-powdered leaves**				
*Avijor*	Cross-validation	0.971	0.994	99.0%
	**Test set Validation**	**1.000**	**0.973**	**97.8%**
*Guara*	Cross-validation	0.829	0.977	95.2%
	**Test set Validation**	**0.867**	**0.987**	**96.7%**
*Isabelona*	Cross-validation	0.914	0.943	93.8%
	**Test set Validation**	**0.933**	**0.987**	**97.8%**
*Marta*	Cross-validation	1.000	0.989	99.0%
	**Test set Validation**	**1.000**	**1.000**	**100%**
*Pentacebas*	Cross-validation	0.886	0.983	96.7%
	**Test set Validation**	**0.933**	**1.000**	**98.9%**
*Soleta*	Cross-validation	0.800	0.994	96.2%
	**Test set Validation**	**1.000**	**1.000**	**100%**
**Fresh leaves**				
*Avijor*	Cross-validation	0.857	0.977	95.7%
	**Test set Validation**	**1.000**	**1.000**	**100%**
*Guara*	Cross-validation	0.829	0.943	92.4%
	**Test set Validation**	**0.600**	**0.987**	**92.2%**
*Isabelona*	Cross-validation	0.686	0.949	90.5%
	**Test set Validation**	**0.867**	**0.907**	**90.0%**
*Marta*	Cross-validation	0.914	0.983	97.1%
	**Test set Validation**	**1.000**	**1.000**	**100%**
*Pentacebas*	Cross-validation	0.886	0.971	95.7%
	**Test set Validation**	**0.867**	**0.133**	**97.8%**
*Soleta*	Cross-validation	0.743	0.960	92.4%
	**Test set Validation**	**0.933**	**0.960**	**95.6%**

Concerning the dried-powdered leaf results, *Avijor* and *Guara* showed the same results as those found by using the entire spectra. Therefore, the removed wavenumbers did not contain important information for the discrimination study. *Isabelona* showed a small decrease of the classification accuracy in the cross-validation with respect to the results obtained using the entire spectrum (93.8% and 96.2%, respectively). This difference was not reflected in the classification accuracy of the test set. *Marta* class showed 100% of accuracy in the test set classification, and a more accurate result in the cross-validation compared to that of the model using all the variables. *Pentacebas* and *Soleta* showed a lower classification accuracy in the cross-validation compared to the ones obtained in the previous model. However, more accurate results were obtained for the test set classification for both varieties. Specifically, the classification accuracy of *Pentacebas* increased from 97.8% to 98.9%. *Soleta* achieved 100% of classification accuracy. In general, the PLS-DA results improved slightly when performing variable selection. The variables removed not only did not provide useful information for varietal differentiation, but their presence increased the complexity of the multivariate calibration models.

Concerning the fresh leaf results, *Avijor* and *Guara* classes showed the same results as when using the entire spectra. The classification accuracy of *Isabelona* increased in the cross-validation compared to that of the previous model, but the result in test set validation was lower, 90.0% of classification accuracy in contrast to 92.2% obtained by using the entire spectra. *Soleta* also showed a greater classification accuracy in the cross-validation, but the same classification rate was obtained in both models. In the cross-validation, *Marta* and *Pentacebas* classes showed lower classification accuracy compared to the results obtained using the entire spectra. However, the classification accuracy of *Marta* in the test set validation was similar to that in the previous model. In contrast, a lower accuracy was found in the test set validation, 95. 6% of classification accuracy by using the selected variables compared to 100% obtained by using all the variables. In general, the PLS-DA results with variable selection were lightly worse than the ones obtained using the whole spectra, which is in contrast with the results achieved in the model with dried-powdered leaves. This can be due to the water content in the fresh leaves, which produces broad spectral bands that can hide other spectral features and decrease the effectivity of the variable selection. The usefulness of variable selection depends then of the type of sample, and the shape of the spectra seems to have an especial influence. In the case of dried-powdered leaves, the raw spectra show more peaks than fresh leaves due to the absence of water, and this seems to favor the discrimination of the varieties.

## Conclusions

The results of this study demonstrated that NIR spectroscopy has the capability to discriminate *Prunus dulcis* varieties with a notable accuracy, up to 100%. The correct classification rate was higher by using dried-powdered leaves than that by using fresh leaves, which was mainly due to the presence of water in fresh leaves. In addition, the sample homogeneity in the dried-powdered leaves may improve the classification rates. However, three varieties reached 100% of classification accuracy in the fresh leaf model, which demonstrated that fresh leaves could also be used in classification studies. Moreover, selecting the most relevant variables allowed reducing the complexity of the data set and increased the accuracy of the model for dried-powdered leaves. In contrast, the classification rate in the model for fresh leaves was better using the whole spectra. This information could be used in other studies of vegetal species discrimination. The main limitations were the genetic proximity of the varieties and the water influence in the fresh samples.

This is the first study that represents an advance in the research and implementation of the NIRS technology in the nursery plant industry as a varietal discrimination tool. Future work will focus on new classification models in order to improve the results obtained in the present study and to develop a deep comparison with the biomolecular techniques.
